# Enhanced ROBO4 is mediated by up‐regulation of HIF‐1α/SP1 or reduction in miR‐125b‐5p/miR‐146a‐5p in diabetic retinopathy

**DOI:** 10.1111/jcmm.14369

**Published:** 2019-05-15

**Authors:** Qiaoyun Gong, Jia'nan Xie, Ying Li, Yang Liu, Guanfang Su

**Affiliations:** ^1^ Eye Center The Second Hospital of Jilin University Changchun Jilin China; ^2^ Department of Ophthalmology Shanghai General Hospital (Shanghai first people hospital), Shanghai Jiaotong University Medical School Shanghai China

**Keywords:** diabetic retinopathy, hypoxia‐inducible factor‐1α, microRNA, roundabout 4, specificity protein 1

## Abstract

Retinal cell damage caused by diabetes leads to retinal microvascular injury. Roundabout 4 (*ROBO4*) is involved in angiogenesis, which varies with the development of diabetic retinopathy (DR). Here, we explored the transcriptional regulation and microRNA‐mediated modulation of *ROBO4* expression and related retinal cell function in DR. A streptozotocin‐induced type I diabetic animal model was established to detect the expression of hypoxia inducible factor‐1α (HIF‐1α), specificity protein 1 (SP1) and ROBO4. Retinal pigment epithelium (RPE) cells were cultured under hyperglycaemia or hypoxia and used for mechanistic analysis. Furthermore, roles of *miR‐125b‐5p* and *miR‐146a‐5p* were evaluated, and their targets were identified using luciferase assays. The cell functions were evaluated by MTS assays, permeability analysis and migration assays. The development of DR increased the levels of HIF‐1α, SP1 and ROBO4 both in the DR model and in hyperglycaemic/hypoxic RPE cells. They were co‐expressed and up‐regulated in diabetic retinas and in RPE cells under hyperglycaemia/hypoxia. Knockdown of HIF‐1α significantly inhibited SP1 and ROBO4, whereas SP1 down‐regulation abolished ROBO4 expression in RPE cells under hyperglycaemia/hypoxia. *miR‐125b‐5p* and *miR‐146a‐5p* were down‐regulated by hyperglycaemia and/or hypoxia. Up‐regulation of miRNAs reversed these changes and resulted in recovery of target gene expression. Moreover, luciferase assays confirmed *miR‐125b‐5p* targeted *SP1* and *ROBO4*, and *miR‐146a‐5p* targeted *HIF‐1α* and *ROBO4* directly. The decreased cell viability, enhanced permeability, and increased cell migration under DR conditions were mitigated by knockdown of HIF‐1α/SP1/ROBO4 or up‐regulation of *miR‐125b‐5p*/*miR‐146a‐5p*. In general, our results identified a novel mechanism that *miR‐125b‐5p*/*miR‐146a‐5p* targeting HIF‐1α/SP1‐dependent ROBO4 expression could retard DR progression.

## 1 INTRODUCTION

Diabetic retinopathy (DR), a microvascular complication of diabetes mellitus (DM), is the leading cause of blindness in adults worldwide.^1^ DR is primarily caused by the long‐term detrimental effects of high glucose,^2^ leading to retinal microvascular defects and neuroretinal dysfunction and degeneration.^3^, ^4^ Notably, high glucose in early‐stage DR can induce increased retinal vessel permeability, leakage of harmful substances and degeneration of capillaries, resulting in retinal hypoxia in the late stage of DR.^5^, ^6^ Hyperglycaemia and hypoxia may interact, contributing significantly to the progression of DR.^7^ The blood‐retinal barrier (BRB), which is comprised of the retinal vasculature and the retinal pigment epithelium, eliminates the neural elements of the retina as well as the cytotoxic products to protect the retina to modulate its extracellular chemical composition.^8^ Endothelial cells harbouring tight junctions are responsible for maintaining the inner BRB, and their impairment results in enhanced vascular permeability.^9^ The outer BRB, which is formed by retinal pigment epithelial (RPE) cells, plays a crucial role in fluid balance within the retina.^10^ Breakdown of BRB resulted from the disruption of tight junctions is the main factor responsible for oedema and neovascularization. Therefore, protecting or reversing retinal microvascular dysfunction is fundamental for many studies of DR. However, extensive work has been carried out to identify factors involved in the disruption of the inner BRB during DR, the mechanisms implicated in outer BRB regulation have been poorly uncovered. As the molecular mechanisms underlying the pathogenesis of DR has not been fully understood, this study will focus on the role of RPE cells in DR.

Roundabout 4 (ROBO4) is specifically expressed in vascular endothelial cells and is involved in angiogenesis and the maintenance of blood vessel stability.^11^, ^12^ ROBO4 maintains the vascular integrity^13^ and promotes pathological angiogenesis through various signalling pathways.^14^, ^15^ *ROBO4* is also strongly overexpressed in the vessels of various types of tumours.^11^, ^16^ In retinal researches, ROBO4 expression and distribution have been studied in the fibrovascular membranes (FVMs) of patients with proliferative DR. *ROBO4* is also expressed in the retinal pigment epithelium (RPE), playing important roles in RPE functions under hypoxia.^17^ Thus, ROBO4 may have a role in the formation of FVMs and could exert physiologic effects on retinal cells. We previously showed that ROBO4 is co‐expressed with hypoxia‐inducible factor‐1α (HIF‐1α) in vessels of FVMs and is positively regulated by HIF‐1α.^18^ HIF‐1α is an oxygen‐sensitive transcription factor that is associated with angiogenesis during the progression of DR and FVM development.^19^, ^20^ Under conditions of low oxygen, hypoxia‐induced proteins are up‐regulated.^19^, ^21^, ^22^ Thus, hundreds of proteins related to cell proliferation, survival, and angiogenesis can be activated by HIF‐1α signalling pathways.^23^ However, the modulatory effects of HIF‐1α on ROBO4 expression are not direct.

Specificity protein 1 (SP1) and HIF‐1α cooperate to promote tumour progression^24^ and activate genes related to cell adaption for hypoxia. Transcriptional regulation of SP1 by HIF‐1α was found to have protective functions in neurotoxicity.^25^ Additionally, SP1 is necessary for full basal expression of ROBO4 in macrovascular endothelial cells.^26^ DNA methylation of the proximal promoter of ROBO4 inhibits SP1 binding, inducing low ROBO4 expression in non‐endothelial cells.^27^ Thus, aberrant levels of ROBO4 induced by HIF‐1α may be mediated via SP1 in DR.

MicroRNAs (miRNAs) are small non‐coding RNAs that play important roles in the progression of DR. miRNAs modulate gene expression through transcriptional or post‐transcriptional mechanisms, inducing mRNA degradation or protein regression by binding to the 3′‐untranslated region (UTR) of target genes.^28^, ^29^

Here, we assessed the roles of *miR‐125b‐5p* and *miR‐146a‐5p* in HIF‐1α/SP1‐mediated ROBO4 expression in vivo in diabetic rats or in vitro in RPE cells under hyperglycaemia or hypoxia.

## 2 MATERIALS AND METHODS

### 2.1 Animal experiments

All animal experiments were conducted in accordance with the NIH Guide for the Care and Use of Laboratory Animals and approved by the Ethics Committee of the Second Hospital of Jilin University.

Male Sprague‐Dawley rats (~200 g, 8 weeks old) were obtained from Animal Center, College of Basic Medical Sciences, Jilin University. They were housed in standard plastic rodent cages and maintained in a controlled environment (24°C, 12‐hours light, 12 hours dark cycle). Diabetes was induced by a single intraperitoneal injection of streptozotocin (STZ; Sigma, St. Louis, MO, USA; 65 mg/kg, in citrate buffer, pH 4.5). Control rats received an identical volume of citrate buffer. Rats were considered diabetic when their blood glucose exceeded 16.7 mmol/L at 72 hours and 1 week after STZ administration. Body weights of rats were also monitored throughout the study. Control or diabetic rats were maintained for 4, 6 and 8 weeks (n = 8/group). Four eyeballs from four rats in each group (NC and DM) were excised for preparation of retinal tissue sections (6 μm) to perform immunofluorescent staining. Retinal tissue for protein analysis and mRNA extraction was conducted in six eyes from each group.

### 2.2 Cell culture and treatments

ARPE‐19 cells were obtained from the American Type Culture Collection (ATCC, Manassas, VA, USA) and maintained in Dulbecco's modified Eagle's medium (DMEM)/F‐12 (Hyclone, China) containing 10% foetal bovine serum (FBS; Gibco, USA). Cultures of ARPE‐19 cells were maintained at 37°C in a humidified atmosphere of 5% CO_2_. For hyperglycaemic studies, the cells were plated at 2,500 cells/cm^2^ in 6‐well plates (Corning, Acton, MA, USA) and treated with normal glucose (NG, 5.5 mmol/L D‐glucose) or high glucose (HG, 25 mmol/L D‐glucose) or with the mannitol osmotic control (MN, 19.5 mmol/L mannitol together with NG) for 1, 3, 5, or 7 days. For hypoxic experiments, cells were plated in 30‐mm dishes (Corning); when the cell confluence reached 70%‐80%, the medium was changed, and cells were plated in a sealed and anaerobic workstation (Ruskin Technologies, Pencoed, Wales, UK) with 1% O_2_, 5% CO_2_ and 94% N_2_, and the same temperature (37°C) and humidity (90%) as that under hypoxic conditions for various times (4, 8, 16 and 24 hours). For hyperglycaemic and hypoxic study, cells were treated with NG or HG for 104 hours, and following by hypoxia and hyperglycaemia for 16 hours. All experiments were conducted at least three times.

### 2.3 Total RNA isolation and identification

Total RNA was extracted from ARPE‐19 cells cultured under different conditions and tissues using an Eastep Super Total RNA Extraction Kit (Promega, Shanghai, China) following the manufacturer's protocol. The concentration and purity of RNA were measured using a NanoDrop 2000c Spectrophotometer (Thermo Fisher Scientific, Waltham, MA, USA). An A260/A280 value of approximate 2.0 was generally accepted for further analysis. The integrity of RNA samples was assessed by 1% agarose gel electrophoresis.

### 2.4 Quantitative analysis of mRNAs and miRNAs

For analysis of mRNA, 500 ng total RNA was reverse transcribed into cDNA using a Perfect Real Time RT reagent kit (Takara Bio, Dalian, China) in a 20‐μL reaction volume. The qPCR mixture contained 1 μL cDNA, 10 μmol gene‐specific primers (forward and reverse mixed together) and 10 μL of 2 × Fast SYBR Green Master Mix (Roche Diagnostics, Switzerland). Three replicates for each biological mixture were analysed on a LightCycler 480 (Roche Diagnostics). The data were normalized to the expression of housekeeping genes according to our previous study.^30^ Specific primers were designed and verified in our previous studies.^30^, ^31^ Primer sequences are listed in Table S1.

To analyse miRNA levels, 800 ng total RNA was polyadenylated and reverse transcribed. The cDNA was diluted 1:5 for further RT‐qPCR with specific primers using TransScript Green miRNA Two‐Step qRT‐PCR SuperMix (Transgen Biotech, Beijing, China). Three replicates were performed for each biological replicate. The mature miRNA sequences were as follows: hsa‐miR‐125b‐5p (MIMAT0000423), 5′‐UCCCUGAGACCCUAACUUGUGA‐3′; hsa‐miR‐146a‐5p (MIMAT0000449), 5′‐UGAGAACUGAAUUCCAUGGGUU‐3′. The quantitative PCR primers against mature miRNAs (cat. nos. HmiRQP0096 and HmiRQP0196) were purchased from GeneCopoeia (Rockville, MD, USA), and the expression levels of miRNAs were normalized to that of U6 expression.

In analysis of miRNAs or mRNAs, two negative controls were included with water instead of template. The relative expression levels of miRNAs or mRNAs were calculated by the 2^−ΔΔCt^ method, which was based on the ratio of gene expression between an experimental and control group.

### 2.5 Western blotting

For in vitro experiments, total protein was collected from ARPE‐19 cells cultured under different conditions. For in vivo experiments, the retinal tissues from control or diabetic rats were isolated and washed in phosphate‐buffered saline (PBS) twice. The cells or tissues were lysed for 60 minutes on ice in Total Protein Extraction Buffer with protease inhibitor (Transgen Biotech) according to the manufacturer's protocol and then sonicated. The lysates were centrifuged at 12,000 rpm for 10 minutes at 4°C. Protein concentrations were determined using a Bicinchoninic Acid Protein Assay Kit (Beyotime, Jiangsu, China). Supernatant proteins were collected, denatured, separated by sodium dodecyl sulphate‐polyacrylamide gel electrophoresis (SDS‐PAGE) with 5% stacking gels and 10% separating gels, and transferred to polyvinylidene difluoride membranes. The membranes were blocked in 5% skim milk for 1 hour and incubated with primary antibodies against HIF‐1α (1:200 dilution; Affinity, USA), SP1 (1:150 dilution; Santa Cruz Biotechnology, Santa Cruz, CA, USA), Robo4 (1:50 dilution; Abcam, Cambridge, MA, USA), ZO‐1 (1:500 dilution; Affinity, USA), Occludin (1:500 dilution; Affinity, USA), Claudin‐1 (1:200 dilution; Affinity, USA) and β‐actin (1:1000 dilution; CMC‐TAG, USA) at 4°C overnight. Membranes were then incubated with secondary antibodies (1:5000; Boster, China) for 40 minutes. Finally, an Enhanced Chemiluminescence (ECL) Plus kit (Millipore, USA) was applied for visualization. The grey bands were calculated using Image J software.

### 2.6 Immunofluorescence

Immunofluorescent staining of HIF‐1α, SP1 and Robo4 was performed using OCT‐embedded sections or cultured ARPE‐19 cells on polylysine‐coated glass coverslips after culture under hyperglycaemic conditions. Slides were rinsed, and cells were fixed with 4% paraformaldehyde, permeabilized with 0.5% Triton X‐100 in PBS for 15 minutes at room temperature, and blocked (1% bovine serum albumin in PBS) for 30 minutes at 37°C. Subsequently, sections were treated with primary antibodies at 4°C overnight, including rabbit polyclonal anti‐HIF‐1α (1:50 dilution; Affinity), rabbit polyclonal anti‐SP1 (1:100 dilution; Affinity), and rat monoclonal anti‐Robo4 antibodies (1:50 dilution; Santa Cruz Biotechnology). Slides were then incubated with Cy3‐conjugated goat anti‐rabbit (Bioss, Beijing, China) or goat anti‐mouse IgG DyLight 488‐conjugated secondary antibodies (Thermo, IL, USA) for 1 hour at 37°C. Nuclei were stained with DAPI (1:600 diluted in PBS; Solarbio, Beijing, China). The slides were observed under a fluorescent illumination microscope (Olympus IX71, Tokyo, Japan).

### 2.7 Transfection with miRNA mimics or small interfering RNA (siRNA)

ARPE‐19 cells in the logarithmic growth phase were seeded in 6‐well plates and cultured under high glucose for 2 days prior to transfection. After reaching 70%‐80% confluence, cells was transfected with 50 nmol miRNA (miR‐125b‐5p or scramble mimic) or 100 pmole siRNA (SP1 siRNA, Robo4 siRNA, or NC siRNA) using Lipofectamine RNAiMAX transfection reagent (Invitrogen, Carlsbad, CA, USA). Five hours after transfection, fresh high‐glucose medium was replaced. After transfection for 48 hours, cells were harvested for further mRNA analysis. After another 24 hours, cells were collected for protein analysis. miR‐125b‐5p and scramble mimics were chemically synthesized by GenePharma (Shanghai, China). The sequence of miR‐125b‐5p mimic was 5′‐UCCCUGAGACCCUAACUUGUGA‐3′, and that of the scramble miRNA was 5′‐UUCUCCGAACGUGUCACGUTT‐3′. The following siRNA sequences were synthesized by Bioneer (Korea): SP1 siRNA, 5′‐CUAUGAACUACAGGUGUUU‐3′; Robo4 siRNA, 5′‐CCUCAGAGUUCACGGACAU‐3′ and negative control siRNA, 5′‐AGUCUCCACGUGUACGUTT‐3′.

For hypoxic studies, at 24 hours after passaging, cells were transfected with 50 nmol miRNA (miR‐146a mimic or scramble) or 100 pmol siRNA (HIF‐1α siRNA, Robo4 siRNA, or NC siRNA) using Lipofectamine RNAiMAX. Cells were treated with hypoxic conditions for 16 hours before mRNA or protein extraction. The sequence of the miR‐146a‐5p mimic was 5′‐UGAGAACUGAAUUCCAUGGGUU‐3′, and that of HIF‐1α siRNA was 5′‐CTGGACACAGTGTGTTTGA‐3′.

### 2.8 Luciferase reporter assay

Luciferase constructs were generated by ligating oligonucleotides containing the wild‐type (WT) or mutant (MUT) putative target site of the HIF‐1α, SP1 or Robo4 3′‐untranslated region (3′‐UTR) into the multiple cloning site of the pmirGLO vector (Promega, Madison, WI, USA). Constructs were verified by sequencing. Human embryonic kidney 293 (HEK293) cells were cultured in 24‐well plates at 2.5 × 10^5^ cells/well in DMEM/F‐12 (Hyclone) supplemented with 10% FBS (Gibco) without antibiotics for 24 hours. HEK293 cells were cotransfected with WT constructs, MUT constructs or vector and miRNA mimics or scramble miRNA using Lipofectamine 2000 (Invitrogen). After transfection for 5 hours, the medium was replaced with fresh medium containing 10% FBS. After 48 hours, luciferase assays were conducted using the Dual‐Glo Luciferase Assay System (E2920; Promega), following the manufacturer's recommended protocol. Light emission was measured using a GloMax 96 Microplate Luminometer (Promega). Firefly luciferase activity was normalized to that of Renilla luciferase for each sample.

### 2.9 Cell viability assay

Cell viability was assessed by 3‐(4,5‐dimethylthiazol‐2‐yl)‐5‐(3‐carboxy‐ methoxyphenyl)‐2‐(4‐sulfophenyl)‐2H‐tetrazolium (MTS; Promega) according to the manufacturer's protocol. ARPE‐19 cells were cultured under hyperglycaemic or hypoxic conditions with transfections as described above and then seeded at a density of 2 × 10^3^ cells/well in 96‐well plates. Before detection, 100 μL fresh medium was replaced, and 20 μL MTS was added to cells. After incubation for 1.5 hours at 37°C under normal oxygen, the absorbance was measured at 490 nm using Varioskan Flash (Thermo).

### 2.10 Monolayer permeability assay

For permeability assays, ARPE‐19 cells treated with different conditions were seeded at 1 × 10^5^ cells/well in the upper chamber (6.5‐mm diameter transwell with 0.4‐μm pore polycarbonate membrane inserts; Corning) and cultured for 48 hours to reach confluence. The upper chamber was washed three times with PBS and treated with FITC‐dextran (1 mg/mL; Sigma). The fluorescence intensity, equivalent to the relative amount of FITC‐dextran in the lower chambers of the transwells, was measured over a 30‐minutes incubation at 37°C and determined in triplicate using Varioskan Flash (excitation wavelength, 490 nm; emission wavelength, 520 nm; Thermo).

### 2.11 Cell migration assay

The migratory ability of ARPE‐19 cells under hyperglycaemic or hypoxic conditions was determined using the transwell system. A total of 5 × 10^3^ cells from each group were seeded in the top chambers of 6.5‐mm diameter transwells with 8.0‐μm pore polycarbonate membrane inserts (3422; Corning) and cultured in 200 μL medium with 5% FBS. Bottom chambers were filled with 500 μL medium with 20% FBS. After incubation for 24 hours, cells on the top chamber were removed, and migrated cells were fixed with 4% paraformaldehyde and stained in 0.1% crystal violet solution. Images were captured, and cells were counted in five fields at 10 × magnification. Quantification was performed using Image J software.

### 2.12 Statistical analysis

Data are presented as means ± standard deviations from at least three independent experiments. One‐way analysis of variance was used for multiple comparisons and followed by the Student‐Newman‐Keuls post‐hoc test to assess the statistical differences between groups. Comparisons between two groups were made using two‐tailed Student's *t* tests (GraphPad Prism 6.0; GraphPad Prism, San Diego, CA, USA). Two‐sided *P* < 0.05 were considered statistically significant.

## 3 RESULTS

### 3.1 HIF‐1α, SP1 and ROBO4 were up‐regulated concomitantly in the retinas of diabetic animals

**Figure 1:**
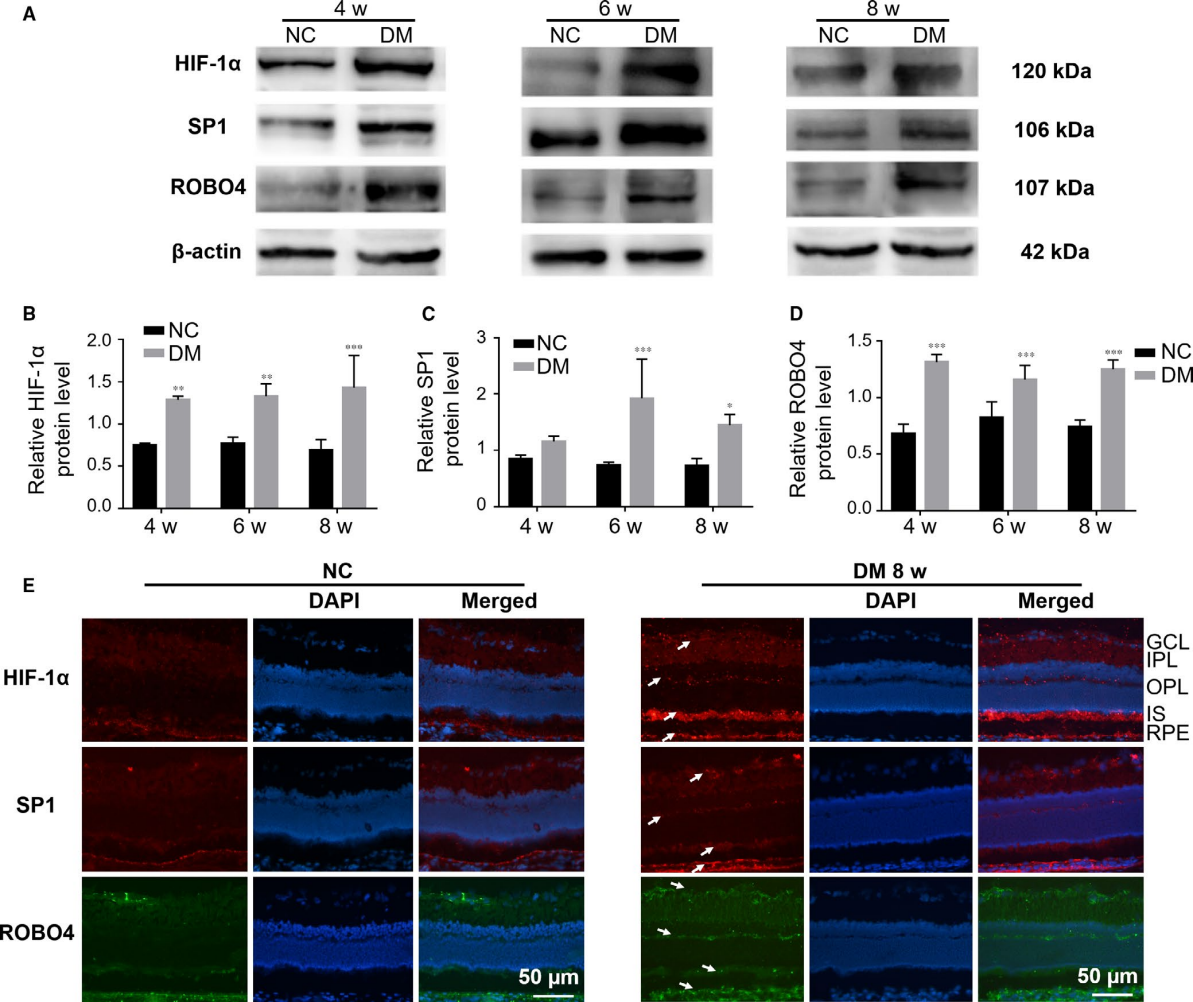
The expression levels of HIF‐1α, SP1 and ROBO4 were increased in the diabetic retina. A‐D, Western blots of HIF‐1α, SP1 and ROBO4 expression in the retinas of diabetic rats after 4, 6 and 8 weeks. β‐Actin was used as a loading control. Bars, mean ± SDs. *P < 0.05; **P < 0.01; ***P < 0.001 versus the respective negative control group (n = 6). E, Immunofluorescent staining in normal or diabetic retinas and nuclear staining by DAPI (magnification: 20×). HIF‐1α (red), SP1 (red) and ROBO4 (green) showed enhanced fluorescence (arrows) in NFL, GCL, IPL, OPL, IS and RPE layers in the diabetic group compared with normal rats. NFL: nerve fibre layer, GCL: ganglion cell layer, IPL: inner plexiform layer, OPL: outer plexiform layer, IS: inner segment, RPE: retinal pigment epithelium

To confirm variations in HIF‐1α, SP1 and ROBO4 expression in the development of early DR, we used an in vivo STZ‐induced diabetic rat model. Blood glucose levels were markedly elevated in STZ‐treated rats during the first week after injection. These DM rats also exhibited significant weight loss that persisted over the course of DR progression as compared with age‐matched control rats (Table S2). Western blotting showed high levels of HIF‐1α, SP1 and ROBO4 after 4 weeks of uncontrolled diabetes, with levels sustained at 6 and 8 weeks (Figure 1A–D). HIF‐1α and ROBO4 were up‐regulated beginning at 4 weeks, whereas SP1 tended to increase at week 4 and was significantly overexpressed at week 6.

Immunofluorescence analysis (Figure 1E) showed that HIF‐1α and SP1 were weakly expressed in all layers of the normal retina, particularly the nerve fibre layer (NFL), ganglion cell layer (GCL), inner plexiform layer (IPL) and RPE. In the retinas of 8‐week DM rats, HIF‐1α and SP1 were up‐regulated not only in the NFL, GCL, IPL and RPE but also in the outer plexiform layer (OPL) and inner section (IS). ROBO4 was weakly expressed in the NFL, GCL, IPL, IS and RPE in normal retina, but up‐regulated in the same layers as well as the OPL in DM rats. Thus, HIF‐1α, SP1 and ROBO4 were co‐expressed and with stronger expression in diabetic retinas than in the normal retina.

### 3.2 HIF‐1α, SP1 and ROBO4 were induced and miR‐125b‐5p was inhibited by hyperglycaemia in ARPE‐19 cells

**Figure 2:**
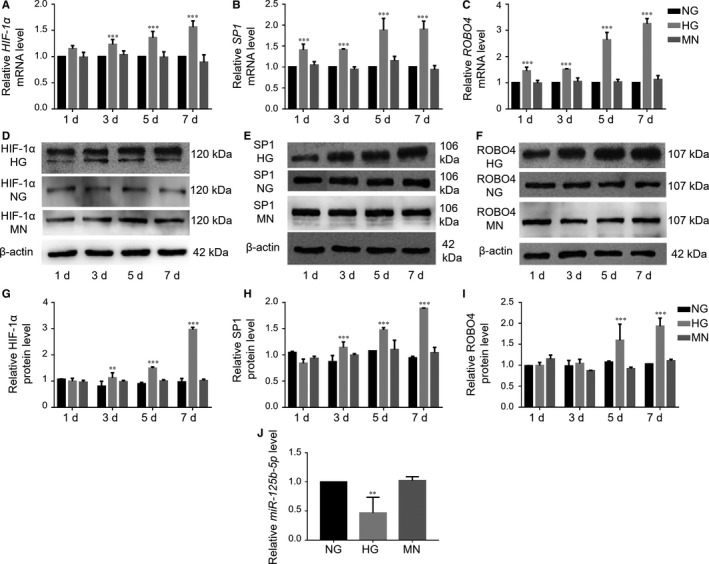
Hyperglycaemia caused enhanced expression of HIF‐1α, SP1 and ROBO4 and down‐regulation of *miR‐125b‐5p* in ARPE‐19 cells. A, *HIF‐1α*, B, *SP1* and C, *ROBO4* mRNA levels under hyperglycaemic conditions compared with the normal glucose (NG) group. There were no significant changes in the mannitol (MN) group. Expression was expressed as a ratio to HPRT‐1, normalized to NG and calculated by the 2^−ΔΔCt^ method. D‐I, Protein levels of HIF‐1α, SP1 and ROBO4 were determined by Western blotting analysis, and the densitometric analysis was normalized to β‐actin expression. J, Determination of *miR‐125b‐5p* expression in ARPE‐19 cells cultured in high glucose (HG) for 5 days using RT‐qPCR, normalized to RNU6B. All groups, n = 3; *P < 0.05; **P < 0.01; ***P < 0.001 versus the respective NG

Next, we explored the alterations in HIF‐1α, SP1 and ROBO4 expression in vitro using ARPE‐19 cells. Notably, high glucose enhanced *HIF‐1α*, *SP1* and *ROBO4* mRNA levels in a time‐dependent manner, whereas mannitol did not induce any obvious changes (Figure 2A–C). Increases in these three genes of more than 1.5‐fold were observed in the hyperglycaemic group at 5‐7 days. Changes in HIF‐1α, SP1 and ROBO4 protein levels were consistent with corresponding mRNA levels (Figure 2D–I). Thus, subsequent experiments of hyperglycaemia were performed using 5‐day treatment with high glucose.

We further investigated whether *miR‐125b‐5p*, which modulates cell differentiation, growth and development in the retina,^32^ was involved in regulating SP1 and ROBO4 in ARPE‐19 cells. TargetScan and mirna.org predicted that SP1 and ROBO4 may be potential targets of *miR‐125b‐5p*. RT‐qPCR analysis of RNA samples from ARPE‐19 cultured under hyperglycaemia for 5 days showed significant *miR‐125b‐5p* down‐regulation (Figure 2J). SP1 and ROBO4 were up‐regulated, and *miR‐125b‐5p* was down‐regulated in ARPE‐19 under hyperglycaemic conditions. Thus, up‐regulation of SP1 and ROBO4 may be mediated by down‐regulation of *miR‐125b‐5p*.

### 3.3 HIF‐1α, SP1 and ROBO4 were co‐expressed and up‐regulated in ARPE‐19 cells under hyperglycaemic conditions

**Figure 3:**
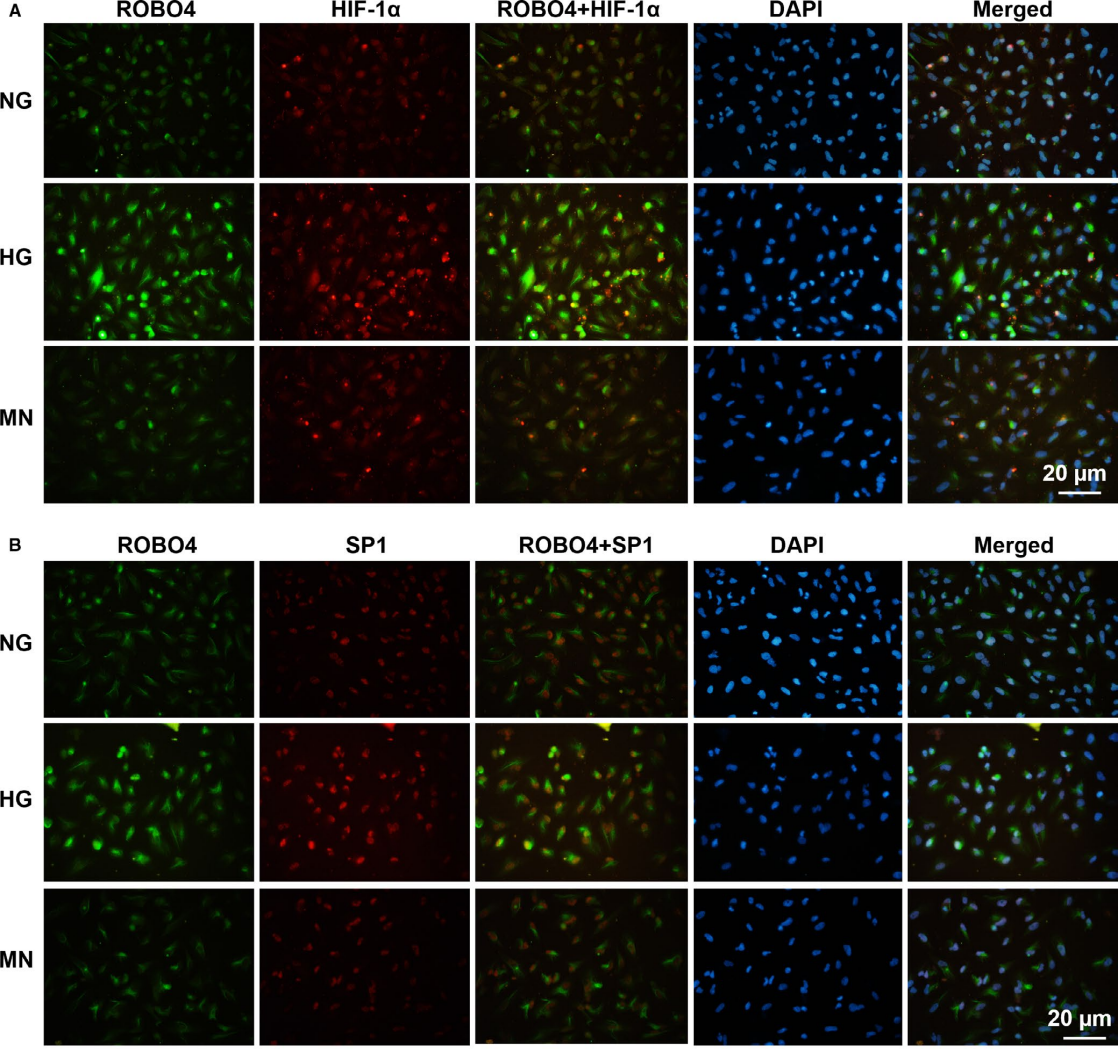
Abundance and localization of HIF‐1α, SP1 and ROBO4 in ARPE‐19 cells under hyperglycaemic conditions. Dual immunostaining of HIF‐1α (red) and ROBO4 (green) A or SP1 (red) and ROBO4 (green) B, merged with DAPI (blue) in RPE cells under NG, HG and MN

Double immunofluorescence staining of high‐glucose‐treated ARPE‐19 cells showed that HIF‐1α was weakly detected in the nucleus and cytoplasm of RPE cells under NG or MN and was strongly enhanced under HG (Figure 3A). ROBO4 showed weak immunofluorescence in the cytoplasm and cytomembrane of RPE cells under NG or MN, and up‐regulation was observed after treatment with HG. SP1 was expressed in the nucleus of RPE cells, with up‐regulation observed after culture in HG (Figure 3B). Co‐staining with ROBO4 showed strong fluorescence in the cytoplasm and cytomembrane of RPE cells in HG compared with that in NG or MN. These results confirmed the localization and up‐regulation of HIF‐1α, SP1 and ROBO4 in RPE cells exposed to high glucose.

### 3.4 Hypoxia induced up‐regulation of HIF‐1α, SP1 and ROBO4 and down‐regulation of miR‐146a‐5p in ARPE‐19 cells

**Figure 4:**
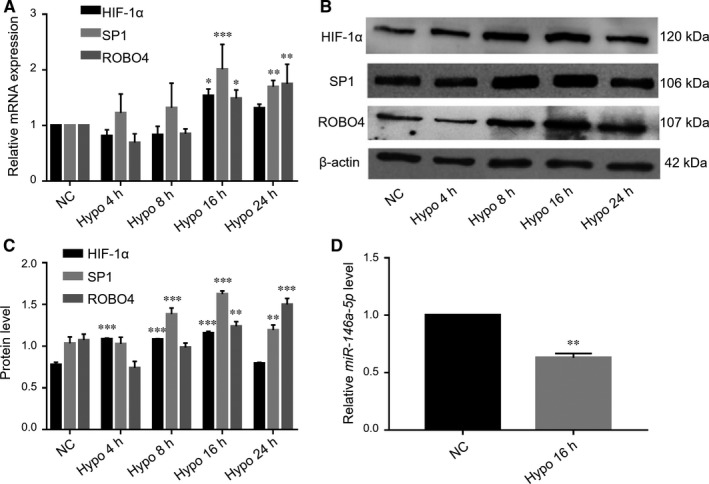
Elevated levels of HIF‐1α, SP1 and Robo4 and decreased expression of *miR‐146a‐5p* in ARPE‐19 cells under hypoxic conditions. A, Relative mRNA levels of *HIF‐1α*, *SP1* and *ROBO4*, expressed as a ratio to *PPIA*, normalized to the normal control group from three separate experiments. Western blotting B, and quantification C, of HIF‐1α, SP1 and ROBO4 normalized to β‐actin expression. D, Hypoxia‐mediated alterations in *miR‐146a‐5p* expression were determined by RT‐qPCR, normalized to RNU6B. All groups, n = 3; *P < 0.05; **P < 0.01; ***P < 0.001 versus the respective negative control group

We then investigated the detailed changes in HIF‐1α, SP1 and ROBO4 during different stage of DR using ARPE‐19 cells under hypoxic conditions. As shown in Figure 4A, *HIF‐1α* mRNA levels decreased during the first 8 hours of hypoxia and then increased. Similarly, *ROBO4* transcripts first decreased and then increased significantly. However, *SP1* was up‐regulated immediately after exposure to hypoxia. In contrast, HIF‐1α protein levels were initially enhanced after exposure to hypoxia and then decreased slightly at 24 hours (Figure 4B and 4C). This discrepancy between HIF‐1α mRNA and protein suggested that HIF‐1α protein may be regulated by changes to protein stability and accumulation.^33^ SP1 and ROBO4 protein levels were consistent with corresponding transcript levels. Hyperglycaemia is the initial cause of DR, resulting in pathological changes in the microvasculature and inducing hypoxia in the retina; hyperglycaemia and hypoxia may then interact and contribute to the progression of DR.^7^ Accordingly, changes in HIF‐1α, SP1 and ROBO4 in ARPE‐19 cells under hypoxia were similar to those under high‐glucose conditions. Additionally, when RPE cells were exposed to hypoxia for 16 hours, HIF‐1α, SP1 and ROBO4 levels remained high compared with the normoxic group. Thus, subsequent experiments of hypoxic conditions were carried out with incubation for 16 hours.

We then analysed the expression of another specific miRNA, *miR‐146a‐5p*, which may target the 3′‐UTRs of HIF‐1α and ROBO4 directly. Notably, *miR‐146a‐5p* has dual roles in nuclear factor‐κB (NF‐κB)‐mediated inflammatory pathways in DR.^34^ This miRNA is transactivated by NF‐κB and exerts negative feedback on NF‐κB activation. *miR‐146a‐5p* overexpression has protective effects on high‐glucose‐treated endothelial cells and retinas of STZ‐induced diabetic rats.^35^ However, the role of *miR‐146a‐5p* in RPE cells under diabetic conditions has not been investigated. Our results showed that *miR‐146a‐5p* was down‐regulated in RPE cells exposed to hypoxia for 16 hours (Figure 4D), and HIF‐1α and ROBO4 were elevated, suggesting that *miR‐146a‐5p* and HIF‐1α/ROBO4 may interact in ARPE‐19 cells.

As high glucose and hypoxia are the two main initiators relating to DR, we performed parallel experiments on RPE cells exposed to a combined insult of high glucose and hypoxia. Then, the expression levels of miR‐125b‐5p and miR‐146a‐5p were detected. Not surprisingly, miR‐125b‐5p and miR‐146a‐5p were found decreased by 2‐fold in RPE cells induced by hyperglycaemia and hypoxia (Figure S1). These results confirmed that miR‐125b‐5p and miR‐146a‐5p were down‐regulated in different stages of DR, and regulation on them may ameliorate the progression of DR.

### 3.5 HIF‐1α promoted ROBO4 expression by regulating SP1 in ARPE‐19 cells under hyperglycaemic or hypoxic conditions

**Figure 5:**
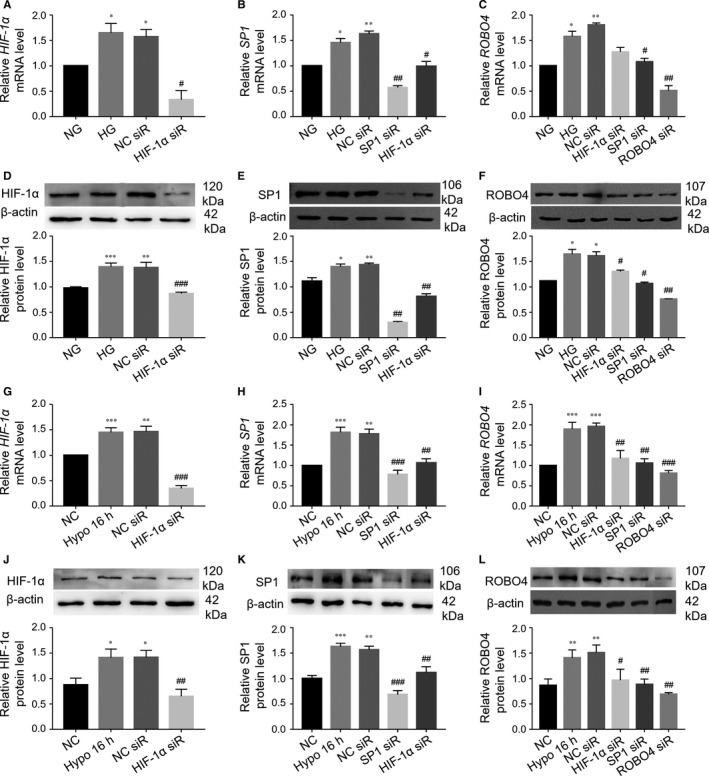
Knockdown of HIF‐1α could block the increase in ROBO4 expression through SP1 in ARPE‐19 cells under hyperglycaemic or hypoxic conditions. A‐C, siRNA transfection to knockdown HIF‐1α, SP1 and ROBO4 in ARPE‐19 cells under HG. HPRT‐1 was used as a reference gene. D‐F, Western blot analysis after siRNA transfection. G‐L, ARPE‐19 cells were transfected with siRNA under hypoxic conditions. The mRNA analysis was based on the ratio to PPIA. β‐Actin was used as a loading control in all Western blot experiments. All groups, n = 3; *P < 0.05; **P < 0.01; ***P < 0.001 versus the respective negative control group. ^#^ *P* < 0.05; ^##^ *P* < 0.01; ^###^ *P* < 0.001 versus the negative control siRNA transfection

To explore the modulatory relationship of HIF‐1α, SP1 and ROBO4 in DR, siRNA was used to silence the expression of these genes. Notably, continuous HG treatment significantly up‐regulated *HIF‐1α*, *SP1* and *ROBO4* mRNAs, with no distinct differences between HG and the negative transfection group (Figure 5A–C). The HG‐induced > 1.5‐fold increases in *HIF‐1α*, *SP1* and *ROBO4* mRNAs were suppressed by transfection with specific siRNAs, showing decreases in more than 2‐fold, lower than that in the NG group. Furthermore, SP1 was repressed with HIF‐1α down‐regulation, and ROBO4 was inhibited following suppression of HIF‐1α or SP1. The suppressive effects of specific siRNAs on hyperglycaemia‐induced HIF‐1α, SP1, and ROBO4 up‐regulation were also observed by Western blotting (Figure 5D–F).

Transfection of ARPE‐19 cells under hypoxia with siRNA targeting HIF‐1α, SP1 or ROBO4 (Figure 5G–L) resulted in >2‐fold decreases in the corresponding target. Notably, SP1 was inhibited by reduction in HIF‐1α expression, and ROBO4 was decreased by down‐regulation of HIF‐1α or SP1 under hypoxia. These data implied that HIF‐1α could positively modulate ROBO4 expression by mediating SP1 in DR models.

### 3.6 miR‐125b‐5p targeted SP1 and ROBO4 directly in ARPE‐19 cells under hyperglycaemic conditions

**Figure 6:**
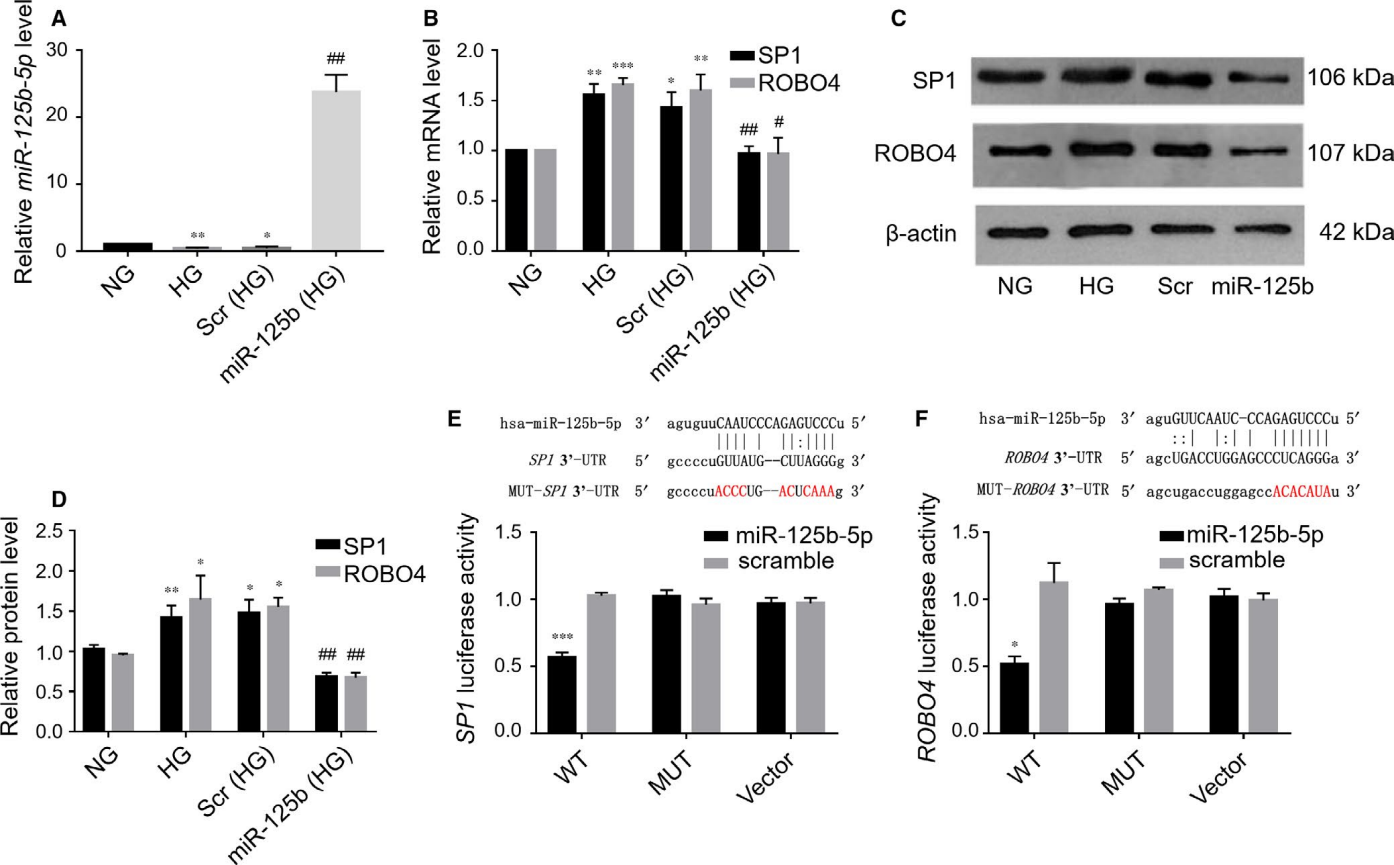
SP1 and ROBO4 were validated as targets of *miR‐125b‐5p*. A, Confirmation of *miR‐125b‐5p* mimic transfection in ARPE‐19 cells under hyperglycaemic conditions by RT‐qPCR quantification compared with miRNA scramble (scr) transfection. miRNA levels were expressed as a ratio to RNU6B. B, Quantitative analysis of *SP1* and *ROBO4* mRNA levels in ARPE‐19 cells under HG following transfection with *miR‐125b‐5p* mimic or a scramble control. Results were normalized to HPRT‐1 and are presented as values relative to the control group. C, D, Western blots and quantification of SP1 and ROBO4 in ARPE‐19 cells under HG following transfection with *miR‐125b‐5p* mimic. β‐Actin was used as a loading control. Scr, scramble control; *miR‐125b*, *miR‐125b‐5p* mimic. All groups, n = 3; *P < 0.05; **P < 0.01; ***P < 0.001 versus NG; ^#^ *P* < 0.05; ^##^ *P* < 0.01 versus scr. E, F, Base‐pair comparison between mature *miR‐125b‐5p* and the WT or MUT putative target site in the 3′‐UTR of *SP1* or *ROBO4* mRNA. The mutated binding site used for the luciferase assay is marked in red. Hsa‐, human. Luciferase activity with various reporters was detected in the presence or absence of *miR‐125b‐5p* mimic in HEK293 cells (n = 3), *P < 0.05; ***P < 0.001 versus MUT plasmid or vector. WT, wild‐type; MUT, mutant

We then explored the functional significance of hyperglycaemia‐induced down‐regulation of *miR‐125b‐5p*. After transfection of ARPE‐19 cells with *miR‐125b‐5p* mimic under HG conditions, *miR‐125b‐5p* was increased by nearly 25‐fold compared with miRNA scramble transfection (Figure 6A). Moreover, this transfection reduced hyperglycaemia‐induced SP1 and ROBO4 up‐regulation at both mRNA and protein levels (Figure 6B–D). No effects were observed in the scrambled miRNA group. Luciferase assays using the wild‐type (WT) or mutant (MUT) version of the predicted binding region in the SP1 or Robo4 3′‐UTR (Figure 6E, 6F) revealed that cotransfection of HEK293 with the corresponding 3′‐UTR WT plasmid and *miR‐125b‐5p* mimic led to a significant decrease in luciferase activity compared with the scrambled mimic. In contrast, *miR‐125b‐5p* mimic did not suppress the luciferase activity of the MUT plasmid or vector. These results confirmed the direct down‐regulation of SP1 and ROBO4 by *miR‐125b‐5p* in ARPE‐19 cells under HG conditions.

### 3.7 Hypoxia‐induced HIF‐1α and ROBO4 expression was inhibited by miR‐146a‐5p in ARPE‐19 cells

**Figure 7:**
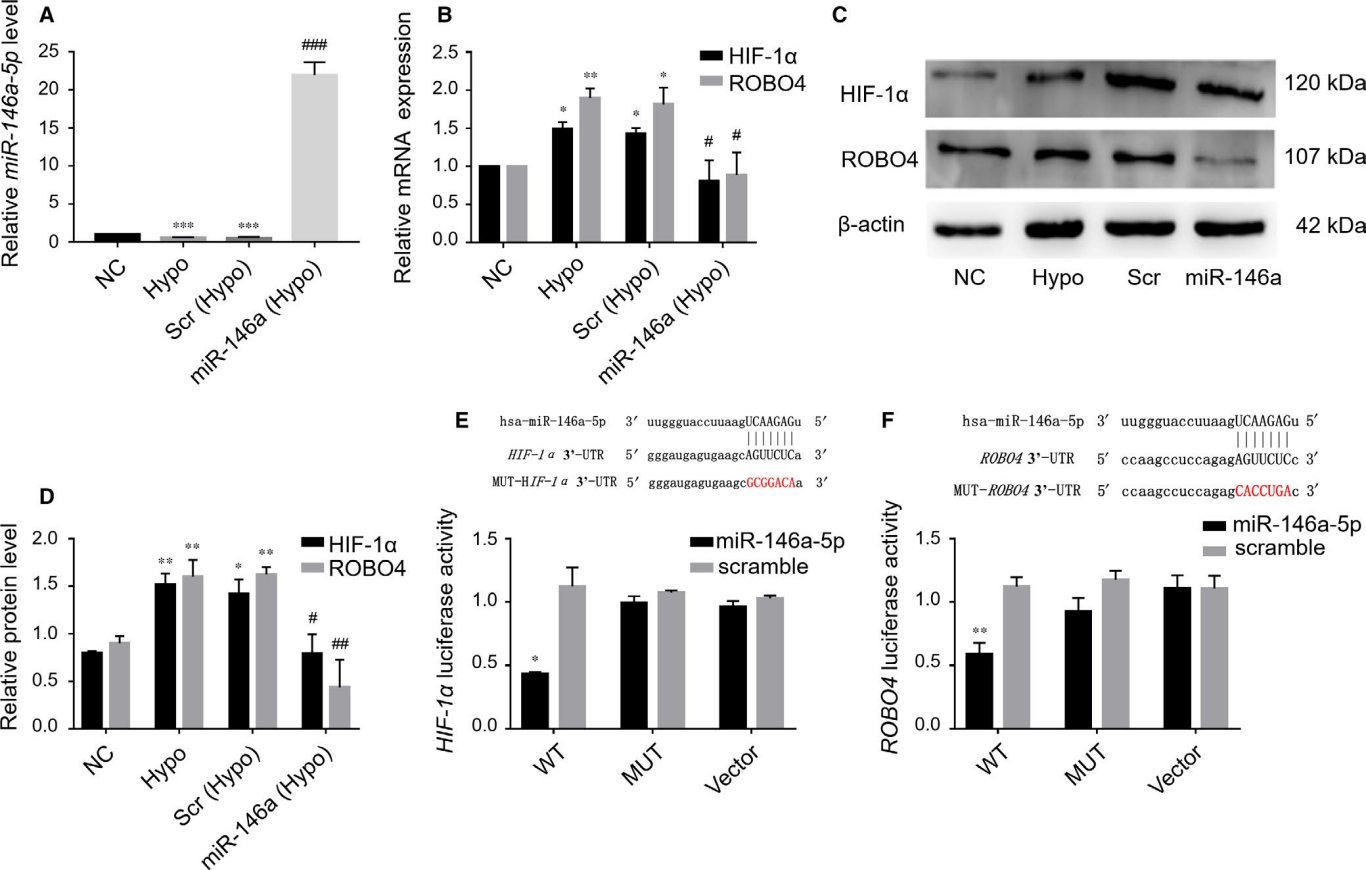
*miR‐146a‐5p* regulated HIF‐1α and ROBO4 directly in ARPE‐19 cells under hypoxic conditions. A, *miR‐146a‐5p* expression in ARPE‐19 cells following exposure to hypoxia and transfection with *miR‐146a‐5p* mimic or scramble mimic (scr). miRNA levels were expressed as the ratio to RNU6B. B‐D, HIF‐1α and ROBO4 mRNA and protein levels in ARPE‐19 cells under hypoxic conditions with transfection with *miR‐146a‐5p* or scr. mRNA levels were expressed as the ratio to PPIA. Protein levels were relative to β‐actin. Scr, scramble control; *miR‐146a*, *miR‐146a‐5p* mimic. All groups, n = 3; *P < 0.05; **P < 0.01; ***P < 0.001 versus NC; ^#^ *P* < 0.05; ^##^ *P* < 0.01; ^###^ *P* < 0.001 versus scr. E, F, Alignment of *HIF‐1α*‐3′‐UTR and *ROBO4*‐3′‐UTR (and mutated 3′‐UTR, red) sequences with mature *miR‐146a‐5p* based on bioinformatics predictions. Luciferase reporter assays in HEK293 cells, showed binding of *HIF‐1α*‐3′‐UTR or *ROBO4*‐3′‐UTR with *miR‐146a‐5p* (n = 3). *P < 0.05; **P < 0.01 versus MUT plasmid or vector. WT, wild‐type; MUT, mutant

Next, we examined the relationships among HIF‐1α and ROBO4 up‐regulation, hypoxia and *miR‐146a‐5p* down‐regulation. *miR‐146a‐5p* was up‐regulated nearly 20‐fold by *miR‐146a‐5p* mimic compared with that in cells transfected with scrambled miRNA (Figure 7A). *miR‐146a‐5p* overexpression blocked hypoxia‐induced HIF‐1α and ROBO4 up‐regulation (Figure 7B–D), whereas no such inhibition was observed in the scrambled mimic group. To confirm direct binding of *miR‐146a‐5p* with the 3′‐UTR of *HIF‐1α* or *ROBO4* mRNA, luciferase assays were performed. HIF‐1α 3′‐UTR or Robo4 3′‐UTR luciferase activity was significantly suppressed with *miR‐146a‐5p* overexpression compared with the scramble mimic (Figure 7E, 7F). However, this suppression was not observed in the mutated or vector groups. Thus, *miR‐146a‐5p* could directly inhibit the increased levels of HIF‐1α and ROBO4 in ARPE‐19 cells under hypoxic conditions.

### 3.8 Down‐regulation of ROBO4 through transcriptional repression or miRNA targeting improved cell functions in ARPE‐19 cells under hyperglycaemic or hypoxic conditions

**Figure 8:**
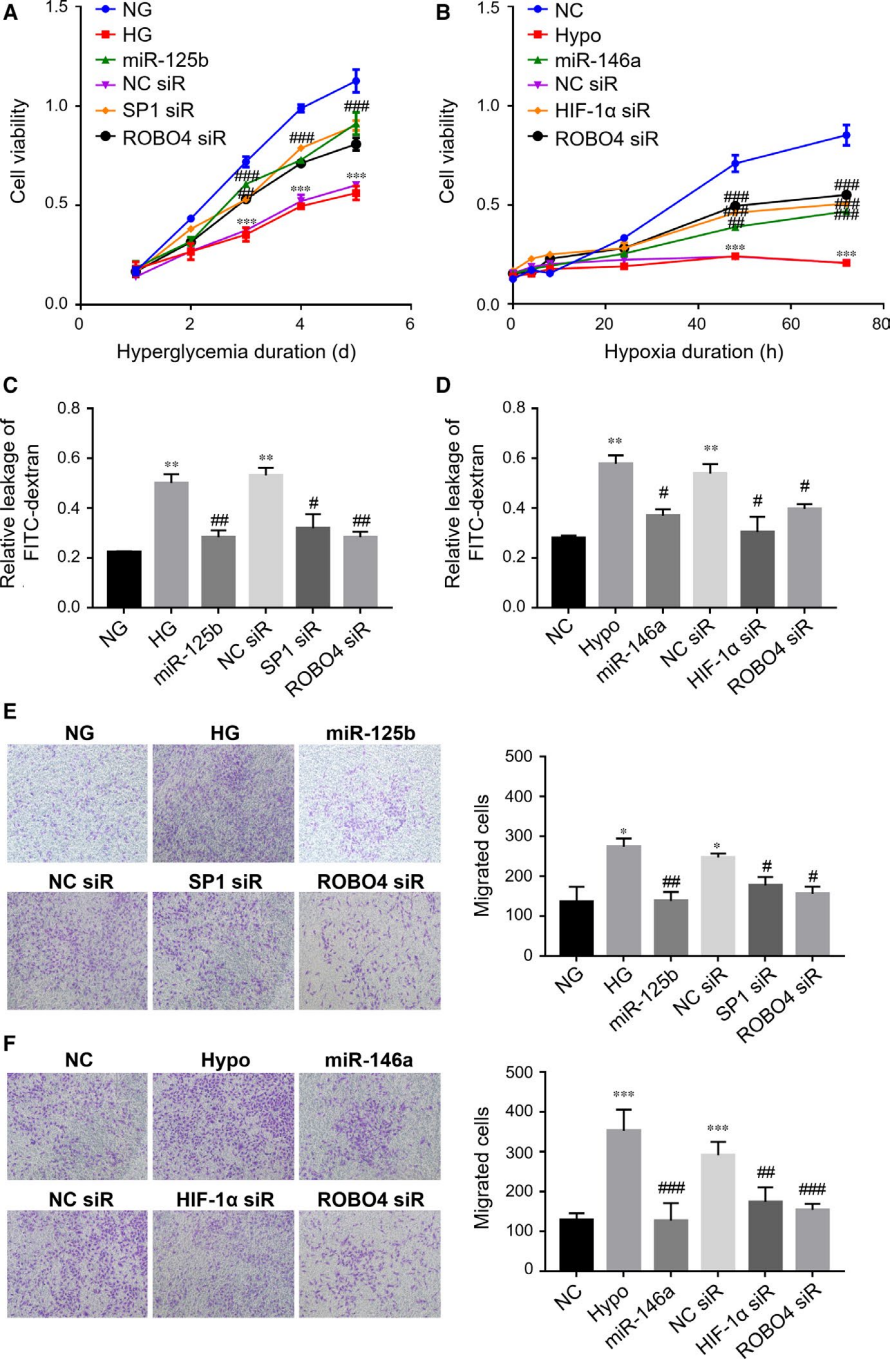
Effects of ROBO4 depletion by transcriptional regulation or miRNA modulation on ARPE‐19 cell viability, monolayer permeability and migration under hyperglycaemic and hypoxic conditions. A, Changes in the viability of ARPE‐19 cells under HG following inhibition of SP1 or ROBO4 or up‐regulation of *miR‐125b‐5p* over time. B, Changes in the viability of ARPE‐19 cells under hypoxic conditions following knockdown of HIF‐1α or ROBO4 or overexpression of *miR‐146a‐5p* over time. C, D, Permeability was analysed by detection of the leakage of FITC‐dextran across cells in monolayer culture under hyperglycaemic or hypoxic conditions following transfection with siRNA or miRNA mimic. E, F, Representative photomicrographs showed the migrated cells in transwell assays. Migrated cells were counted at a magnification of 10×. NG, normal glucose. In HG experiments, *P < 0.05; **P < 0.01; ***P < 0.001 versus NG. In hypoxic studies, **P < 0.01; ***P < 0.001 versus NC; ^#^ *P* < 0.05; ^##^ *P* < 0.01; ^###^ *P* < 0.001 versus negative transfection (n = 3). Hypo, hypoxia; NC siR, negative transfection

The effects of HIF‐1α, SP1 and ROBO4 expression on RPE cells under DR conditions were then evaluated. RPE cell viability was significantly decreased compared with that in normal glucose or normoxic conditions (Figure 8A, 8B). Transfection with SP1 or ROBO4 siRNA or *miR‐125b‐5p* mimic under HG promoted RPE cell viability when cells were incubated for more than 3 days. Similarly, knockdown of HIF‐1α/ROBO4 by siRNA or *miR‐146a‐5p* elevated the viability of RPE cells under hypoxia compared with that in cells transfected with the negative control. Thus, HIF‐1α, SP1 and ROBO4 had important regulatory roles in RPE cells under hyperglycaemia or hypoxia.

Cell permeability assays demonstrated that hyperglycaemia or hypoxia increased the leakage of FITC‐dextran compared with that in the normal group (Figure 8C, 8D). When exposed to HG, the monolayer permeability of RPE cells was reduced with the presence of SP1 or ROBO4 siRNA or *miR‐125b‐5p* mimic compared with that of negative transfection. Under hypoxic conditions, HIF‐1α siRNA, ROBO4 siRNA and *miR‐146a‐5p* could prevent the leakage of FITC‐dextran in RPE cells, while there was no improvement in cells subjected to negative transfection. Thus, these findings indicated that HIF‐1α, SP1 and ROBO4 modulated RPE permeability under high glucose or hypoxia. To further confirm these findings, the expression of tight junction‐related proteins was evaluated. In DM rats, ZO‐1, Occludin and Claudin‐1 were significantly down‐regulated in diabetic retinas compared with that in NC rats at weeks 4, 6, and 8 after STZ administration (Figure S2). Moreover, hyperglycaemia down‐regulated ZO‐1 and Occludin in RPE cells. Low ZO‐1 and Occludin expression in RPE cells under HG could be elevated by SP1 or ROBO4 siRNA or by *miR‐125b‐5p* mimic compared with that of mock transfection (Figure S3). Furthermore, ZO‐1, Occludin and Claudin‐1 were down‐regulated in RPE cells under hypoxic conditions. Knockdown of HIF‐1α or ROBO4 or overexpression of *miR‐146a‐5p* up‐regulated these tight junction‐related proteins in RPE cells exposed to hypoxia (Figure S4)*.* Accordingly, these findings justified that modulation of ROBO4 by HIF‐1α/SP1 knockdown or miRNA targeting had a protective role in RPE cell permeability under diabetic conditions.

Transwell assays revealed that hyperglycaemia or hypoxia significantly promoted cell migration compared with that in normal groups (Figure 8E, 8F). Untransfected cells and negative control cells under hyperglycaemia or hypoxia did not show any significant differences in migration. However, under hyperglycaemia, SP1 siRNA, ROBO4 siRNA and *miR‐125b‐5p* could protect against HG‐stimulated RPE migration, and HIF‐1α siRNA, ROBO4 siRNA and *miR‐146a‐5p* could also prevent hypoxia‐induced RPE migration. Thus, inhibition of SP1/ROBO4 under hyperglycaemia or HIF‐1α/ROBO4 under hypoxia reduced cell motility. Accordingly, knockdown of ROBO4 by upstream inhibition of HIF‐1α/SP1 or miRNA improved the RPE morphology in the progression of DR.

## 4 DISCUSSION

DR is a metabolic disease resulting from long‐term hyperglycaemia, and the pathogenesis of DR is extremely complicated. Biochemical and metabolic abnormalities under hyperglycaemia, together with neuronal dysfunction, lead to increased vascular permeability followed by macular oedema and retinal neovascularization.^1^, ^2^, ^36^ BRB imbalance that result in the leakage of fluids and lipids into the retina contributes to DR progression. It is recognized that the inner BRB is composed mainly of endothelial cells, and the outer BRB is formed by RPE cells; both BRBs are involved in DR progression. Thus, protecting or reversing BRB dysfunction including retinal endothelial cells and RPE cells are crucial for ameliorating DR. However, the role of RPE cells in DR has not been studied extensively. The RPE has recently been shown to play an important role in the progression of DR.^37^ Hyperglycaemia in early stage and hypoxia in late period of DR are two main initiators associated with retinal pathologic changes, which are suggested to be bound up with induction of oxidative stress, inflammation, proliferation, neovascularization and so on. Therefore, here, we examined in vivo STZ‐induced diabetic animals and in vitro RPE cells cultured under hyperglycaemic or hypoxic conditions.

ROBO4 has dual roles in vascular networks,^11^, ^12^, ^13^ and overexpression of ROBO4 inhibits vascular endothelial growth factor (VEGF)‐induced angiogenic signalling and increases vessel maturation in diabetes‐induced cerebral neovascularization,^38^ but it can accelerate the pathological angiogenesis in various tumours. Here, we found that there was a significant increase in ROBO4 expression in the retinas of DM rats. To the best of our knowledge, this was the first time ROBO4 expression was assessed in diabetic retinas. Additionally, in vitro experiments confirmed the increased expression of ROBO4 in ARPE‐19 cells in response to hyperglycaemia or hypoxia and showed that ROBO4 was localized in RPE cells under hyperglycaemia. Enhanced ROBO4 expression caused by the progression of DR promoted cell dysfunction, whereas inhibition of ROBO4 ameliorated these abnormal changes. Interestingly, the role of ROBO4 in modulating the vasculature may be dependent on cell types and context; in microvascular complications in the eye resulting from DM, ROBO4 may play a destructive role in DR progression.

The different roles of ROBO4 in the vascular system may result from its upstream regulation in various diseases. There are multiple SP1 binding sites on the *ROBO4* promoter region, and HIF‐1α can regulate SP1.^25^ Here, we found that SP1‐mediated HIF‐1α modulated ROBO4 in the pathological progression of DR for the first time. Additionally, HIF‐1α, SP1 and ROBO4 levels were elevated concomitantly in vivo in a model of DR and in vitro in RPE cells cultured under hyperglycaemia or hypoxia. Additionally, HIF‐1α, SP1 and ROBO4 co‐expressed in the diabetic retina and in RPE cells under hyperglycaemia. Importantly, HIF‐1α could regulate ROBO4 by affecting the expression of SP1. We recently showed that there are two SP1 binding sites in the promoter region of *ROBO4* in human retinal endothelial cells (HRECs) under hyperglycaemia.^39^ We concluded that the binding site at –1912/–1908 could be induced by hyperglycaemia and played a major role in increasing *ROBO4* transcription in HRECs in the simulated DR environment. The modulating function of SP1 on ROBO4 in RPE cells under hyperglycaemic or hypoxic conditions was also confirmed in this study. Moreover, by down‐regulating ROBO4, knockdown of HIF‐1α or SP1 significantly improved cell viability, mitigated the increased permeability and restricted RPE migratory ability under hyperglycaemic or hypoxic conditions. However, differences in cellular functions mediated by HIF‐1α, SP1 and ROBO4 were observed due to the influence of other target genes, such as VEGF and intercellular adhesion molecule (ICAM‐1), which can be stimulated by HIF‐1α^40^ and SP1.^41^, ^42^ Further validation of the regulatory relationships among HIF‐1α, SP1 and ROBO4 and associated functional experiments in vivo are necessary.

In this study, we identified another level of regulation for DR‐induced HIF‐1α, SP1 and ROBO4 up‐regulation and the biological effects mediated by miRNAs. Accumulating evidence has demonstrated the role of miRNAs in the pathogenesis of diabetes and its complications, including DR.^43^, ^44^ We have determined some differentially expressed miRNAs in the development of DR.^45^ This study further verified that *miR‐125b‐5p* targeted SP1 and ROBO4 directly in RPE cells under hyperglycaemia, whereas *miR‐146a‐5p* was a direct modulator of HIF‐1α and ROBO4 under hypoxia. After the initial identification of glucose and/or hypoxia‐induced *miR‐125b‐5p* down‐regulation and *miR‐146a‐5p* reduction in RPE cells, we used miRNA mimics to identify the biological significance of the miRNAs in vitro. Up‐regulation of *miR‐125b‐5p* blocked the increase in SP1 and ROBO4 levels in RPE cells induced by HG. Similarly, overexpression of *miR‐146a‐5p* inhibited HIF‐1α and ROBO4 up‐regulation in RPE cells exposed to hypoxia, and they had protective roles in cell morphology. Thus, miRNA‐mediated transcriptional modulation of target genes may have a therapeutic role in DR.

Here, it is the first time showing that *miR‐125b‐5p* and *miR‐146a‐5p* could play vital roles in down‐regulating ROBO4 to ameliorate cellular dysfunctions in DR. Interestingly, *miR‐125b* is increased in the vascular smooth muscle cells of diabetic mice and plays a role in the dysregulation of *Suv39h1* and associated chromatin H3K9me3 related to the increased expression of inflammatory genes.^46^ This may be explained by previous studies demonstrating the diverse roles of miRNAs in various physiological and pathological conditions and the associations of miRNAs with many cellular processes, including feedback loops for various signal transduction pathways.^29^, ^34^, ^43^, ^44^ In previous studies, *miR‐146a‐5p* was down‐regulated and its overexpression played a protective role in DR through modulation of fibronectin and NF‐κB, which are involved in inflammatory pathways.^35^, ^47^ Because one miRNA can target multiple genes, more genes associated with the progression of DR have been shown to be regulated by the same miRNA, supporting the potential therapeutic applications of miRNAs.

In this study, DR promoted retinal permeability in diabetic rats and RPE cells under hyperglycaemia or hypoxia. The levels of tight junction‐related proteins were decreased during DR in diabetic retinas, consistent with a previous study,^48^ and supplemented the breakdown of BRB in diabetic retinas. Monolayer permeability assays and analysis of ZO‐1 and Occludin protein levels confirmed the modulatory effects of ROBO4 on the outer BRB under DR conditions. Knockdown of ROBO4 by transcriptional regulation of HIF‐1α/SP1 or *miR‐125b‐5p/miR‐146a‐5p* targeting could reduce the increased monolayer permeability of RPE cells and elevate the decreased tight junction‐related proteins expression induced by hyperglycaemia or hypoxia, similar to the results of previous studies.^49^, ^50^, ^51^ However, Wang et al^52^ showed that HG alone only minimally affected cell permeability, except adding hypoxic conditions. We found that HIF‐1α was up‐regulated after 5 days of hyperglycaemia. Thus, the RPE cells at day 5 under HG were influenced by glucose and oxygen deficiency, which could explain our current findings. In contrast, Villarroel et al^53^ showed that HG reduced RPE cell permeability. However, in their following study, Villarroel et al^54^ found that HG plus IL‐1β induced the increment of permeability and the disruption of ARPE‐19 cell. Overall, HG alone in the initial stage of DR is not the main reason to cause increased permeability, but with the development of DR, other pathophysiological factors contribute to the increment of permeability in RPE cells. Moreover, the decreased viability of RPE cells observed in the study was supported by previous studies.^48^, ^55^ Further studies are needed to elucidate the complex mechanisms regulating RPE permeability and viability. In conclusion, the study demonstrated the regulatory effects of HIF‐1α‐mediated SP1 regulation and miRNAs on ROBO4 expression in the development of DR. Down‐regulation of ROBO4 significantly improved cell functions under different conditions in DR. Therefore, targeting specific miRNAs in combinational therapy may have the potential to prevent the expression of multiple genes in multifaceted diseases, such as DR.

## CONFLICT OF INTEREST

The authors declare no conflict of interest associated with this manuscript.

## AUTHORS' CONTRIBUTION

QG and GS conceived and designed the experiments. QG, JX, YLi and YLiu contributed to the acquisition of data. QG and JX analysed and interpreted the data. QG and GS contributed to drafting the article. All authors have revised the manuscript critically for important intellectual content and approved the final version to be published.

## Data Availability

The datasets used and/or analysed during the current study are available from the corresponding author on reasonable request.
