# Supplementary material for: Enhanced ROBO4 is mediated by up‐regulation of HIF‐1α/SP1 or reduction in miR‐125b‐5p/miR‐146a‐5p in diabetic retinopathy
Source: J Cell Mol Med. 2019 May 15;23(7):4723–37. doi: 10.1111/jcmm.14369 (PMC6584523; doi:10.1111/jcmm.14369)
Supplement: Supplementary file 1 [file JCMM-23-4723-s001.docx]

**Electronic Supplementary Materials**

| Time (week) | | 0 | 1 | 4 | 6 | 8 |
| --- | --- | --- | --- | --- | --- | --- |
| Blood  glucose (mmol/L) | NC | 4.425±0.699 | 5.275±0.465 | 5.200±0.548 | 4.887±0.584 | 4.838±0.481 |
|  | DM | 4.950±0.359 | 24.420±3.701 | 24.640±5.454 | 25.375±1.591 | 24.638±1.905 |
| Weight (g) | NC | 224.25±6.13 | 262.75±7.37 | 354.50±7.19 | 399.63±8.48 | 450.88±8.94 |
|  | DM | 216.20±6.71 | 200.00±29.49 | 229.4±27.29 | 203.25±13.94 | 196.75±6.84 |

Table S1.

Name, primer sequences of genes.

| Gene symbol | Gene name | Primer sequence (5′-3′) |
| --- | --- | --- |
| PPIA | Peptidylprolyl isomerase A | F: AGACAAGGTCCCAAAGAC R: ACCACCCTGACACATAAA |
| HPRT1 | Hypoxanthine phosphoribosyl transferase 1 | F: GACCAGTCAACAGGGGACAT  R: CCTGACCAAGGAAAGCAAAG |
| HIF-1α | Hypoxia induced factor 1-α | F: TACCCACCGCTGAAACGC R: TAGGCTCAGGTGAACTTTGTCT |
| SP1 | Specificity protein 1 | F: TCCAGACCATTAACCTCAGTGC  R: TGTATTCCATCACCACCAGCC |
| ROBO4 | Roundabout4 | F: CCCTGTGCTTGGAACTCAGTG R: CGCTGATGTACCCATAGGTGG |

Table S2.

The blood glucose level and weight of negative control (NC) and diabetes mellitus (DM) rats after the administration of STZ at 0, 1, 4, 6 and 8 weeks.

**Figures**

**Figure. S1.**

**
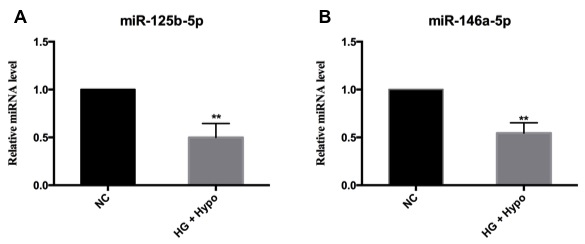
**

**Figure. S1.** Hyperglycaemia and hypoxia induced reduction of miR-125b-5p and miR-146a-5p in RPE cells. (A) miR-125b-5p and (B) miR-146a-5p were inhibited in RPE cells under high glucose for 104 h, and following by hypoxia for 16 h. The miRNA level was normalized to RNU6B (n = 4); **p < 0.01 versus the negative control group.

**Figure. S2.**


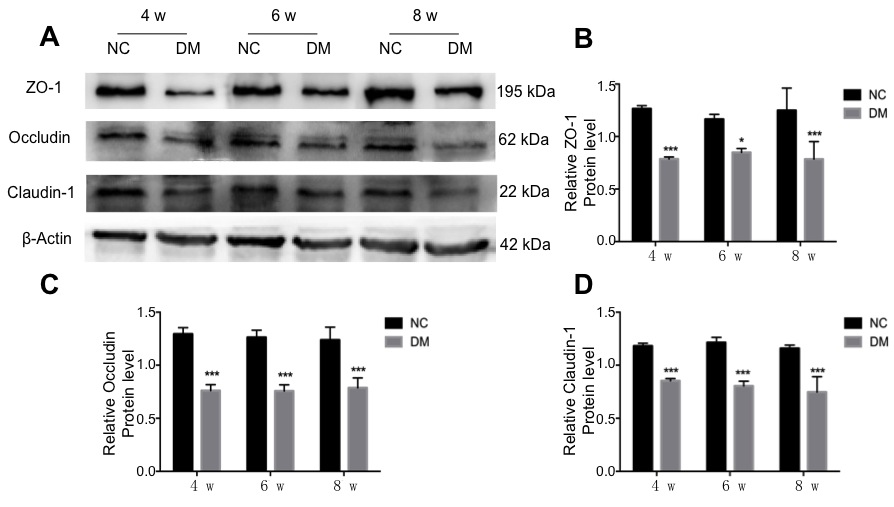


**Figure. S2.**The protein levels of ZO-1, Occludin and Claudin-1 were decreased in the retinas of diabetes mellitus (DM) rats compared with that of normal control (NC) rats after the administration of STZ for 4, 6 and 8 weeks. (A) Western blots of ZO-1, Occludin and Claudin-1 expression in the retinas of NC or diabetic rats after 4, 6, and 8 weeks. β-Actin was used as a loading control. (B-D) The quantified protein levels calculated by Image J. Bars, means ± SDs. *p < 0.05; **p < 0.01; ***p < 0.001 versus the respective NC group (n = 6).

**Figure. S3.**


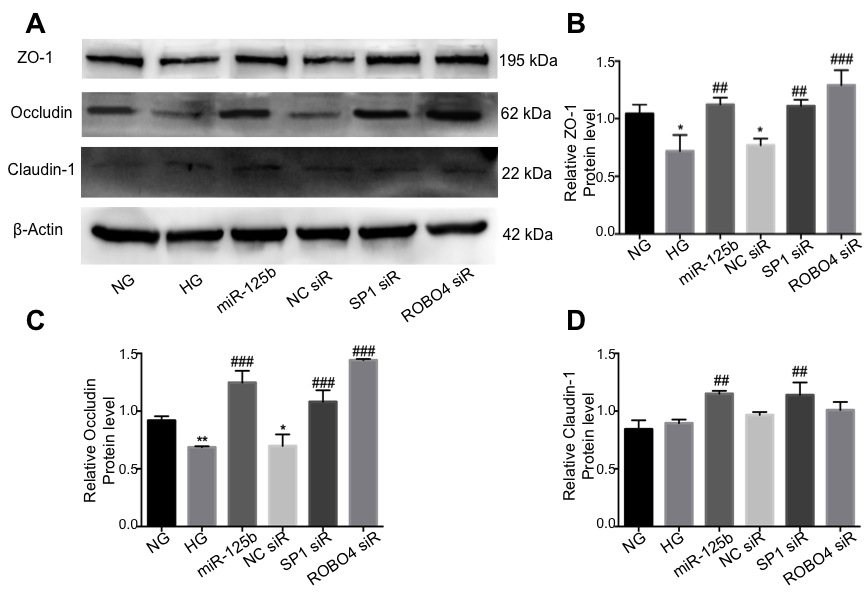


**Figure. S3.** The reduced protein levels of ZO-1 and Occludin induced by hyperglycaemia could be reversed by the transfection of SP1/ROBO4 siRNA or *miR-125b-5p* mimic in ARPE-19. (A) The blots of ZO-1, Occludin and Claudin-1 in ARPE-19 under different conditions. (B-C) The quantified protein levels of ZO-1 and Occludin were decreased significantly under HG, and the knockdown of SP1/ROBO4 and overexpression of *miR-125b-5p* elevated their levels in RPE cells*.* No significant change was observed in the negative transfection group. (D) The expression level of Claudin-1 was not influenced by the hyperglycaemia, while the transfection of *miR-125b-5p* mimic and SP1 siRNA enhanced the level of Claudin-1. β-Actin was used as a loading control. All groups, n = 3; **p* < 0.05; ***p* < 0.01 versus the respective normal glucose (NG) group. ## *p* < 0.01; ### *p* < 0.001 versus the negative transfection.

**Figure. S4.**


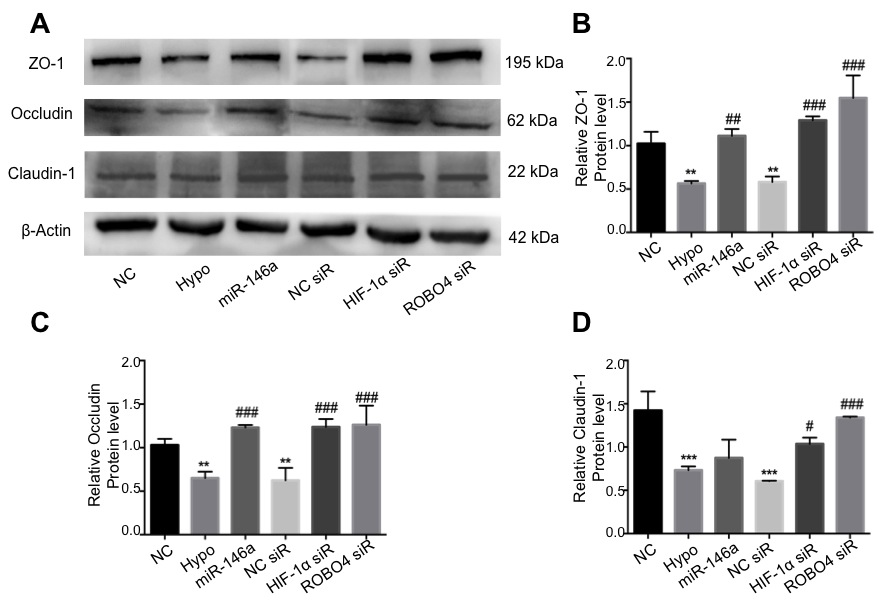


**Figure. S4.** Knockdown of HIF-1α/ROBO4 or upregulation of *miR-146a-5p* enhanced the decreased contents of ZO-1 and Occludin caused by hypoxia in ARPE-19. (A) Western blots of tight junctions-related proteins in RPE cells under hypoxia with transfections. (B-C) The levels of ZO-1 and Occludin were inhibited by hypoxia in RPE cells, while the downregulation of HIF-1α/ROBO4 or overexpression of *miR-146a-5p* can normalise their expression. No significant change was observed in the negative transfection group. (D) The reduced level of Claudin-1 induced by hypoxia could be upregulated by the inhibition of HIF-1α or ROBO4 in ARPE-19, but no significant enhancement was observed by *miR-146a-5p* transfection. β-Actin was used as a loading control. All groups, n = 3; ***p* < 0.01; ****p* < 0.001 versus the respective normal glucose (NG) group. # *p* < 0.05; ## *p* < 0.01; ### *p* < 0.001 versus the negative transfection.
